# Founder Attributes and Self-Reported Decision-Making Styles in Startup Execution: A Dual-Process Perspective on Strategic and Operational Decision Contexts

**DOI:** 10.3390/bs16071130

**Published:** 2026-07-06

**Authors:** Ramesh Menon, Leena James, Elangovan N, Ramesh Chandra Babu T

**Affiliations:** School of Business and Management, Christ University, Hosur Road, Bengaluru 560029, India

**Keywords:** startup decision-making, execution phase, founder attributes, dual-process theory, strategic decisions, operational decisions, entrepreneurial cognition

## Abstract

Problem: Entrepreneurial decision-making is widely recognized as central to startup outcomes, yet how founders make decisions during the startup execution phase remains underexplored. Prior research rarely distinguishes between strategic decisions (e.g., market entry, scaling) and operational decisions (e.g., coordination, problem-solving), even though these two decision types differ in their uncertainty, reversibility, and cognitive demands. Objective: This study investigates how founder attributes relate to self-reported decision-making styles across strategic and operational decision contexts during startup execution. Methodology: Drawing on Dual-Process Theory, decision-making is viewed as an interplay between intuitive (System 1) and analytical (System 2) cognitive processes. A sequential exploratory mixed-methods design was employed, beginning with semi-structured interviews with 20 Indian startup founders to develop the conceptual framework, followed by quantitative examination using Partial Least Squares Structural Equation Modelling (PLS-SEM) on data from 350 funded startup founders, with separate structural models estimated for strategic and operational decision contexts. Results: The findings revealed context-specific patterns of association between founder attributes and self-reported decision-making styles across strategic and operational decision contexts. In the strategic model, cognitive orientation, domain experience, and risk appetite were significantly associated with decision-making style, explaining 49.7% of the variance (R^2^ = 0.497). In the operational model, only risk appetite remained significant, with substantially lower explanatory power (R^2^ = 0.125). Taken together, the findings indicate stronger patterns of association between founder attributes and decision-making style in the strategic context than in the operational context. Conclusions: The study contributes to entrepreneurial cognition research by demonstrating that founder attributes exhibit context-specific patterns of association with decision-making styles. These findings underscore the importance of considering decision context when examining entrepreneurial decision-making.

## 1. Introduction

Startups are widely recognized as engines of innovation, economic growth, and employment generation (Ries, 2011; Blank, 2013). Despite improved access to funding, digital infrastructure, and entrepreneurial support systems, startup failure rates remain persistently high, with estimates suggesting that approximately 90% of new ventures fail within their early years (Startup Genome, 2019). While market misalignment, financial constraints, and competitive pressures are frequently cited as reasons for failure, emerging evidence suggests that these challenges are rooted in founders’ decision-making processes. Consequently, founder decision-making is increasingly recognized as a key determinant of startup success and failure.

Entrepreneurship is inherently decision-driven, situated at the intersection of individuals and opportunities (Shane, 2003; McMullen & Shepherd, 2006). Founders regularly operate in environments characterized by uncertainty, limited information, resource constraints, and time pressure, requiring them to make consequential decisions without the benefit of complete data (Busenitz & Barney, 1997; McVea, 2009). This challenge has only become more complex with the rapid growth of digital and AI-enabled tools capable of processing vast amounts of information to support decision-making. While such technologies have expanded founders’ ability to gather and analyze information, they have not yet eliminated the need for human judgment. Nevertheless, founders remain responsible for interpreting information, evaluating alternatives, and making decisions under uncertainty. Understanding how founders think and make decisions therefore remains highly relevant in contemporary entrepreneurial environments.

A substantial body of research has examined entrepreneurial decision-making from different perspectives, including heuristics and biases, effectuation, causation, entrepreneurial judgment, improvisation, and decision-making under uncertainty (Sarasvathy, 2001; Busenitz & Barney, 1997; McMullen & Shepherd, 2006). These studies have generated important insights into how entrepreneurs recognize opportunities, assess risks, and respond to changing circumstances. Much of the existing literature has focused on opportunity identification, venture creation, and entrepreneurial behavior. Limited research has examined decision-making during the startup execution phase, particularly whether founders adopt different decision-making styles for strategic and operational decisions, and how these styles relate to founder attributes. Unlike traditional small businesses, startups are characterized by evolving business models, greater uncertainty, and ambitions for rapid scaling. Founders routinely make both strategic decisions, such as market entry and fundraising, and operational decisions related to day-to-day execution. These differences suggest that founders may rely on different decision-making styles depending on the decision context.

Dual-Process Theory offers a useful lens for understanding these differences. The theory proposes that individuals use two broad modes of reasoning: an intuitive, fast, and experience-based mode of thinking (System 1), and a slower, more deliberate, and analytical mode of thinking (System 2) (Evans & Frankish, 2009; Kahneman, 2011). Entrepreneurship scholars have long argued that both forms of cognition are important when navigating uncertainty. Yet, limited empirical evidence exists regarding how founders rely on these decision-making styles across different decision contexts during the execution phase, or how founder attributes are associated with these preferences.

To address these gaps, the present study examines how founder attributes such as cognitive orientation, risk appetite, and domain experience relate to self-reported decision-making styles across strategic and operational decision contexts during the startup execution phase. Employing a sequential exploratory mixed-methods design, the study combines qualitative insights from interviews with 20 successful Indian startup founders and quantitative assessment of the proposed relationships using survey data from 350 funded startup founders analyzed through Partial Least Squares Structural Equation Modelling (PLS-SEM).

The study makes three contributions. First, it extends entrepreneurial cognition research by focusing specifically on the execution phase, a stage that has received comparatively less attention than opportunity recognition and venture creation. Second, it highlights the importance of decision context by examining founder attributes and self-reported decision-making styles separately in strategic and operational decision contexts. Third, by applying Dual-Process Theory to startup execution, the study advances a more context-sensitive understanding of entrepreneurial cognition by demonstrating that founder attributes exhibit distinct patterns of association with decision-making styles across decision contexts.

## 2. Literature Review

### 2.1. Entrepreneurship, Startups, and the Centrality of Decision-Making

Entrepreneurship research has evolved from early trait-based explanations toward process-oriented and cognition-driven perspectives. While early studies emphasized personality characteristics and small business behaviour, subsequent research shifted toward opportunity identification, venture creation, and the cognitive mechanisms underlying entrepreneurial action (Shane & Venkataraman, 2000; McMullen & Shepherd, 2006; Davidsson, 2016). This evolution reflects a broader consensus that entrepreneurship is fundamentally a decision-driven process shaped by how individuals interpret uncertainty and act on opportunities.

Within this broader field, startups have emerged as a distinct research context. Unlike traditional small businesses, startups operate under conditions of extreme uncertainty, innovation intensity, and scalability imperatives (Blank, 2013; Ries, 2011). Scholars increasingly argue that startups warrant distinct theoretical treatment because of their non-linear growth trajectories, experimentation-based learning, and high failure rates (Ghezzi & Cavallo, 2020). Despite this recognition, much of the empirical literature continues to generalize findings from small business settings, limiting their applicability to startups.

A growing body of research suggests that factors associated with startup success, including founder traits, execution capabilities, and environmental conditions, may operate through founder decision-making processes rather than directly influencing venture outcomes (Shepherd et al., 2015; Grégoire et al., 2010). However, these factors are often studied in isolation, providing limited insight into how they collectively relate to decision-making during the startup execution phase.

### 2.2. Entrepreneurial Cognition and Decision-Making Under Uncertainty

Entrepreneurial cognition research provides a foundation for understanding how founders process information and make decisions under uncertainty. Entrepreneurs frequently rely on heuristics and cognitive shortcuts due to time pressure and limited information availability (Busenitz & Barney, 1997). While early research often viewed heuristics as a source of systematic bias, more recent scholarship recognizes them as adaptive tools that enable purposeful action in highly uncertain environments (Shepherd, 2020).

Entrepreneurial cognition research encompasses several complementary, though partly overlapping, streams of inquiry. Effectuation and causation theory (Sarasvathy, 2001) distinguishes between founders who work forward from available means and those who work backward from predefined goals. Research on entrepreneurial improvisation examines how founders respond spontaneously to unexpected events and changing circumstances (Hmieleski & Corbett, 2008). Entrepreneurial judgment research views decision-making as action under genuine uncertainty, where outcomes cannot be fully predicted or calculated in advance (Foss & Klein, 2012). Similarly, studies on pivoting and decision-making under uncertainty have explored how founders adapt strategies and revise decisions as new information emerges (McMullen & Shepherd, 2006; Ghezzi & Cavallo, 2020). Although these streams have advanced the understanding of entrepreneurial decision-making, they have largely evolved in parallel, each emphasizing different aspects of how entrepreneurs respond to uncertainty. As a result, limited attention has been given to how founders’ cognitive approaches may vary across different decision contexts during startup execution.

Entrepreneurial decision-making is inherently context-dependent and evolves across venture stages (Shepherd et al., 2015). During the startup execution phase, founders must balance exploration and exploitation while making both strategic and operational decisions. However, much of the existing literature treats decision-making as undifferentiated, overlooking meaningful variation across decision types and contexts. Moreover, existing research is predominantly variance-based, focusing on associations between variables while giving relatively little attention to the cognitive processes through which decisions are formed (Dimov, 2011; McMullen & Shepherd, 2006), thereby limiting our understanding of how founders navigate different decision contexts during startup execution.

### 2.3. Founder Attributes and Decision-Making Processes

Founder-level attributes, including personality traits, cognitive styles, risk appetite, and prior experience, have been widely linked to entrepreneurial behaviour and decision-making. Research based on the Big Five personality framework suggests that traits such as openness to experience, conscientiousness, and emotional stability are positively associated with entrepreneurial engagement and venture performance (Zhao & Seibert, 2006; Brandstätter, 2011; Obschonka et al., 2019). Beyond entrepreneurial outcomes, traits such as conscientiousness, emotional stability, and extraversion have been linked to leadership effectiveness, suggesting that these characteristics may influence how entrepreneurs guide teams and make decisions under pressure (Judge et al., 2002).

Beyond personality traits, entrepreneurial decision-making is influenced by psychological resources that help founders navigate uncertainty and adversity. Research suggests that adaptive coping strategies, such as problem-focused coping and cognitive reappraisal, can support decision quality under uncertain conditions (Carver & Connor-Smith, 2010). Similarly, resilience enables entrepreneurs to recover from setbacks and maintain effective functioning during challenging periods (Luthans et al., 2007). Although these factors are not the primary focus of the present study, they highlight the broader psychological context within which entrepreneurial decisions are made.

Research on cognitive styles suggests that founders differ in how they process information and approach decision-making. Some tend to rely on intuition and pattern recognition, whereas others prefer more deliberate analysis and systematic evaluation when assessing situations and making decisions (Hodgkinson & Sadler-Smith, 2018; Allinson & Hayes, 1996). Risk appetite has been identified as a central driver of entrepreneurial action. Founders with higher risk tolerance are generally more willing to pursue uncertain opportunities, commit resources under ambiguity, and act despite incomplete information (Zhao et al., 2010; Krueger & Dickson, 1994; Markowska & Wiklund, 2020). Similarly, prior domain experience provides knowledge structures that may be associated with how founders approach decision-making and evaluate opportunities (Shane, 2000; Ucbasaran et al., 2008). Collectively, these attributes have been associated with how founders perceive uncertainty and make entrepreneurial decisions.

Despite these contributions, most studies examine founder attributes as direct predictors of entrepreneurial outcomes, with limited attention given to the mechanisms through which these attributes are associated with decision-making processes across different decision contexts. Consequently, limited understanding exists regarding how founder attributes relate to decision-making styles during the startup execution phase.

### 2.4. Decision-Making in Startup Execution: Strategic and Operational Contexts

The startup execution phase presents founders with a complex decision environment in which they must navigate qualitatively distinct decision types, particularly strategic and operational decisions. Classic work on strategic decision processes (Mintzberg et al., 1976) describes strategic decisions as novel, consequential, and complex choices that shape organizational direction, while operational decisions are generally more routine and focused on day-to-day implementation. Similarly, Eisenhardt and Zbaracki (1992) characterize strategic decisions as involving the alignment of organizational resources with a changing environment under conditions of uncertainty, multiple stakeholders, and longer time horizons. In contrast, operational decisions tend to be shorter-term, more structured, and guided by established routines. This distinction is particularly relevant in startups, which operate with evolving business models, limited resources, and heightened uncertainty. Startup founders must simultaneously set strategic direction while managing day-to-day operations. Strategic decisions, such as market entry, fundraising, business model adaptation, and scaling, often involve significant resource commitments and long-term consequences. Operational decisions, by contrast, focus on implementation, coordination, process management, and problem-solving, and are generally narrower in scope and easier to reverse.

Recent startup research highlights that founders frequently alternate between exploratory and execution-oriented activities as ventures evolve, requiring different forms of judgment and information processing (Shepherd & Gruber, 2020). Despite growing interest in entrepreneurial cognition, limited research has examined whether founders employ different decision-making styles across strategic and operational decision contexts. Existing evidence offers mixed perspectives on the role of intuition and analysis in entrepreneurial decision-making. Some studies highlight the adaptive value of intuition in highly uncertain and dynamic environments, where rapid judgments and pattern recognition can facilitate timely action (Mitchell et al., 2004; Hodgkinson & Sadler-Smith, 2018). Other studies emphasize the benefits of analytical reasoning and structured planning, particularly when evaluating alternatives and allocating resources (Delmar & Shane, 2003; Patel & Fiet, 2009). Rather than viewing these findings as contradictory, they may indicate that the effectiveness of intuitive and analytical approaches depends on the nature of the decision being made. Accordingly, distinguishing between strategic and operational decisions provides a useful lens for understanding how founders navigate uncertainty during startup execution and for examining how decision-making styles are associated with different decision contexts.

### 2.5. Dual-Process Theory in Startup Execution Contexts

Dual-Process Theory offers a well-established cognitive framework for understanding decision-making under uncertainty (Kahneman, 2011; Evans & Frankish, 2009). The theory proposes that individuals draw on two broad modes of reasoning: an intuitive, fast, and experience-based mode of thinking (System 1) and a slower, more deliberate, and analytical mode of thinking (System 2). Scholars have widely used this distinction to explain how individuals navigate uncertainty, time pressure, and incomplete information.

Recent developments in Dual-Process Theory suggest that the relationship between intuitive and analytical thinking is more complex than originally proposed. Rather than operating as separate modes of cognition, intuition and reflective reasoning often work together during decision-making. Reflective reasoning enables individuals to evaluate and, when necessary, revise or override their initial intuitive judgments (Byrd et al., 2023; Byrd, 2025). This perspective challenges the traditional view that decision-makers rely predominantly on either intuition or analysis and instead suggests that both forms of reasoning contribute to judgment and choice.

In startup execution contexts characterized by time pressure, resource constraints, and evolving business models, both cognitive systems are likely to be engaged. Intuitive processing enables adaptive responses to dynamic conditions, while analytical reasoning supports structured decision-making in high-uncertainty, high-consequence situations (Hodgkinson & Sadler-Smith, 2018). Emerging evidence indicates that decision effectiveness depends on the alignment between cognitive processing and situational demands.

Despite its relevance, applications of Dual-Process Theory in entrepreneurship have generally treated decision-making as relatively stable across contexts, with limited attention to how the balance between intuitive and analytical reasoning may vary across different types of decisions. This limitation is particularly relevant during the startup execution phase, where founders must navigate both strategic and operational decisions that differ in complexity, uncertainty, and time horizon.

The present study addresses this limitation by examining founders’ self-reported decision-making styles across strategic and operational decision contexts. In doing so, it extends the application of Dual-Process Theory to startup execution and advances a more context-sensitive understanding of entrepreneurial cognition by exploring how founder attributes relate to intuitive and analytical decision-making styles across strategic and operational contexts.

### 2.6. Research Gap and Theoretical Positioning

Despite significant advances in entrepreneurial cognition research, several gaps remain. First, much of the entrepreneurship literature continues to draw on evidence from small business contexts, limiting its applicability to startups characterized by higher uncertainty and more dynamic conditions. Second, while existing research has generated important insights into entrepreneurial cognition, limited attention has been paid to decision-making during the startup execution phase, where founders must continuously balance strategic and operational demands. Third, founder attributes are often examined as direct predictors of entrepreneurial outcomes, providing limited insight into how these attributes relate to founders’ decision-making processes.

A further limitation concerns the treatment of decision-making as a relatively uniform phenomenon. Although strategic and operational decisions differ in scope, uncertainty, and managerial significance, this distinction has rarely been incorporated into empirical studies of entrepreneurial cognition. Consequently, limited evidence exists regarding how founder attributes are associated with decision-making styles across strategic and operational decision contexts.

The present study addresses these gaps by adopting a Dual-Process Theory perspective to examine founders’ self-reported decision-making styles across strategic and operational decision contexts during startup execution. By integrating founder attributes, decision context, and Dual-Process Theory, the study advances a more process-oriented and context-sensitive understanding of entrepreneurial cognition during the startup execution phase.

## 3. Methodology

### 3.1. Research Design

This study adopted a sequential exploratory mixed-methods design (Creswell & Plano Clark, 2018). This approach was appropriate because limited research has examined how founder attributes relate to decision-making styles during the startup execution phase, particularly across strategic and operational decision contexts. Accordingly, the study began with a qualitative phase to develop a deeper understanding of founder decision-making, followed by a quantitative phase to examine the proposed relationships.

The qualitative phase explored how founders make decisions during startup execution and identified the key constructs and relationships underpinning the proposed conceptual framework, which subsequently guided the development of the conceptual model and hypothesis. The quantitative phase then employed a cross-sectional survey design and Partial Least Squares Structural Equation Modelling (PLS-SEM) to examine the proposed relationships across a larger sample of startup founders. To examine context-specific patterns of association between founder attributes and self-reported decision-making styles, two structural models were estimated using the same sample of founders. The first model examined Strategic Decision Style as the outcome variable, while the second examined Operational Decision Style. Each model was interpreted independently to describe the patterns of association observed within its respective decision context. No formal statistical comparison between the models was performed.

The study followed an abductive approach, enabling movement between findings and existing theory, consistent with its pragmatic research orientation. Ethical approval was obtained from the Ethics Committee of Christ University, Bengaluru. Participation was voluntary, and confidentiality and anonymity were assured throughout the study.

### 3.2. Qualitative Phase

#### 3.2.1. Sample and Data Collection

The qualitative phase focused on founders of successful Indian startups, where success was operationalized as either securing external funding (seed to Series B) or achieving sustained profitability for a minimum of two years. This criterion ensured that participants had substantial experience making consequential entrepreneurial decisions under conditions of uncertainty.

A purposive sampling strategy was used, drawing on professional networks, startup ecosystems, and investor connections. Of the 35 founders approached, 20 agreed to participate. Participants represented a diverse range of sectors, venture types, and founder backgrounds. Eleven founders (55%) had prior experience in their startup’s domain, while nine (45%) had entered a new domain. Participants ranged in age from 28 to 52 years, with 60% above the age of 35. Most participants were male (85%), and educational backgrounds ranged from bachelor’s degrees to postgraduate qualifications. The sample included founders from fintech (n = 3), edtech (n = 4), healthtech (n = 3), SaaS/B2B technology (n = 5), and consumer services (n = 5). The ventures ranged in age from 2 to 9 years, and all participants held direct decision-making responsibility as founding members. Additional demographic and venture-level characteristics are presented in Table 1.

Data were collected through semi-structured interviews conducted in person or via video conferencing (Google Meet) between December 2023 and June 2024, each lasting 120–150 min. The interview protocol was informed by prior research on entrepreneurial cognition and decision-making (e.g., Shane, 2003; Grégoire et al., 2010; Alvarez & Barney, 2007; Baron, 2004). The interviews explored four broad areas: (1) entrepreneurial motivation and opportunity recognition, (2) risk perception and risk appetite, (3) decision-making during startup execution, and (4) reflection and cognitive preferences. The interviews explored how founders approached decisions, balanced intuition and analysis, responded to uncertainty, and drew on prior experience when making decisions. All interviews were recorded with consent and transcribed verbatim.

Representative interview questions included: “Can you walk me through how you typically make important business decisions?”; “What factors influence your decisions?”; and “Do you find that your approach changes depending on the type of decision being made?”. Follow-up questions and probes were used extensively to explore specific decision episodes, clarify emerging themes, and capture contextual nuances. Data collection and analysis proceeded iteratively, with interviews continuing until theoretical saturation was reached. Theoretical saturation was defined as the point at which additional interviews no longer generated substantively new insights into founder attributes, decision-making styles, or decision contexts. The complete interview protocol is provided in Appendix A.

#### 3.2.2. Data Analysis

Qualitative data were analysed using grounded theory procedures (Strauss & Corbin, 1998), following a systematic process of open, axial, and selective coding. During the open coding stage, interview transcripts were reviewed line-by-line and segmented into discrete units of meaning. Initial concepts related to entrepreneurial decision-making under uncertainty were identified, including prior domain experience, risk appetite, cognitive orientation, decision context, and intuitive and analytical reasoning. A constant comparative approach was employed, whereby data segments were compared across interviews to refine and consolidate emerging concepts. Coding was conducted manually by the primary researcher and reviewed by a second author. Emerging codes and categories were discussed periodically to refine interpretations and ensure consistency. Where differences in interpretation arose between the primary researcher and the second author, they were resolved through discussion and consensus, with reference to the interview transcripts and the evolving coding framework.

In the axial coding stage, relationships among these concepts were examined to identify recurring patterns and develop higher-order categories. During the selective coding stage, these categories were integrated into an explanatory framework linking founder attributes, decision-making styles, and decision contexts. The resulting framework informed the development of the conceptual model and hypothesis. Theoretical saturation was assessed throughout the coding process and was considered achieved when additional interviews no longer generated substantively new concepts, categories, or relationships. Saturation was reached after the 17th interview, with the remaining three interviews confirming the stability of the coding framework. Table 2 provides a sample coding progression matrix illustrating how representative quotations were developed into open codes, higher-order categories, and selective themes.

### 3.3. Emergent Constructs, Conceptual Framework, and Hypothesis

The qualitative analysis identified three core constructs that help explain decision-making during the startup execution phase. Founder Attributes capture founders’ cognitive and experiential profiles, including cognitive orientation (Mind Type), decision orientation (Tactics Type), risk appetite, domain experience, and area of expertise. Decision Style refers to founders’ self-reported reliance on intuitive (System 1) and analytical (System 2) modes of reasoning. Decision Type captures the distinction between strategic decisions, which are typically high-impact, long-term, and difficult to reverse, and operational decisions, which are more short-term, execution-focused, and routine in nature.

**Figure 1:**
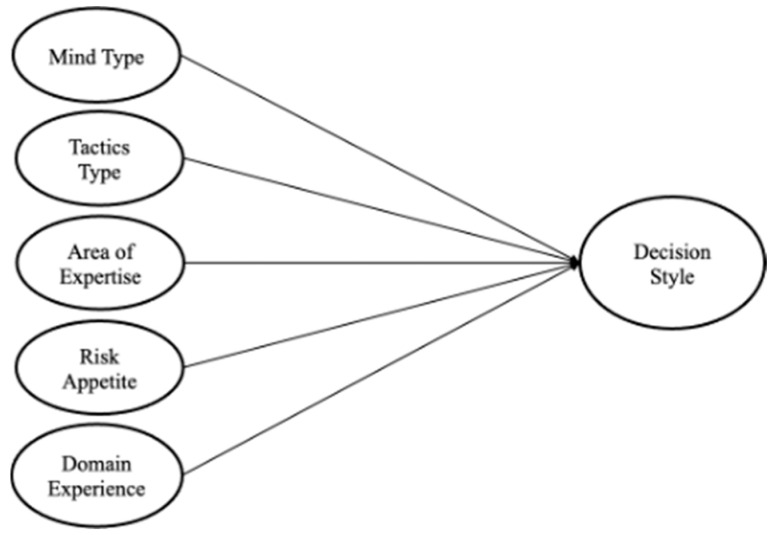
Conceptual Model.

A key insight from the qualitative analysis was that the relationships between founder attributes and self-reported decision-making styles appeared to vary across strategic and operational decision contexts. These findings suggest that decision context is an important consideration when examining entrepreneurial decision-making during the startup execution phase. The resulting conceptual model, which guided the quantitative phase, is presented in Figure 1.

Based on these qualitative insights, the following hypothesis was formulated to guide the quantitative phase:

**H1.**  *Founder Attributes (Mind Type, Tactics Type, Domain Experience, Area of Expertise, and Risk Appetite) are expected to exhibit context-specific patterns of association with self-reported Decision Style across strategic and operational decision contexts.*

### 3.4. Quantitative Phase

#### 3.4.1. Sample and Data Collection

The quantitative phase focused on founders of funded Indian startups. The target population was identified through startup databases, including Inc42 New Delhi India, Tracxn Bengaluru India, and Crunchbase San Francisco USA; and was restricted to startups founded between 2008 and 2018 to ensure that founders had sufficient exposure to the startup execution phase. This resulted in an accessible population of 3436 founders. Based on the sample size guidelines of Krejcie and Morgan’s (1970), a minimum of 341 valid responses was required.

A quota sampling strategy was employed to ensure balanced representation of founders with and without prior domain experience, reflecting the importance of prior domain experience identified in the qualitative phase and incorporated into the conceptual framework. Within each quota group, respondents were selected using non-probability sampling methods, which are commonly used in entrepreneurship research due to the practical challenges of accessing founder populations (Davidsson, 2016). A total of 350 usable responses were retained for analysis. Table 1 presents the demographic and venture-level characteristics of the respondent sample.

Data were collected using a structured online questionnaire administered between September 2024 and February 2025, using email outreach (n = 50), venture capital network channels (n = 250), and telephone follow-ups (n = 50). Participation was voluntary, and respondents were assured that their responses would remain confidential and anonymous throughout the study.

#### 3.4.2. Measures

The survey instrument was developed from the constructs identified in the qualitative phase and aligned with the conceptual framework. All construct items were measured using a five-point Likert-type response scale ranging from 1 (Strongly Disagree) to 5 (Strongly Agree).

Domain Experience was measured using the statement, “I had no prior experience in my startup’s industry/domain before launching it,” such that higher scores indicated lower levels of prior industry experience. Area of Expertise was measured using a self-report item assessing founders’ prior expertise in finance or management acquired through education, professional experience, or specialized training. Although these variables are presented categorically in Table 1 to describe the sample, they were operationalized as self-reported measures in the structural model to capture founders’ perceived experience and expertise. While both variables reflect objective founder attributes, self-report was considered appropriate as founders are well positioned to report their prior experience and expertise accurately.

Risk Appetite was measured using three items capturing founders’ willingness to commit resources under uncertainty, their comfort with ambiguity, and their tendency to pursue uncertain opportunities. Mind Type was measured using four items assessing founders’ preference for intuitive versus analytical information processing. Tactics Type was measured using four items capturing founders’ preferred approach to decision-making and execution, including structured planning, exploration of alternatives, and execution focus. Decision Style was measured separately for strategic and operational decision contexts using four different items for each context. The items assessed founders’ self-reported reliance on analytical and intuitive reasoning when making strategic and operational decisions during the startup execution phase. This approach aligns with the study’s focus on self-reported decision styles rather than direct behavioral measures of cognitive processing.

Following the measurement model assessment, the two items demonstrating the strongest psychometric properties were retained for the Mind Type, Tactics Type, and Decision Style constructs. The instrument was informed by prior research on entrepreneurial cognition, cognitive styles, decision-making, and risk-taking (Allinson & Hayes, 1996; Scott & Bruce, 1995; Zhao et al., 2010), while being adapted to the startup execution context identified in the qualitative phase. To establish content validity, the questionnaire was reviewed by three academic experts and two experienced startup founders. A pilot study involving 20 startup founders was subsequently conducted, resulting in minor wording refinements to improve clarity and contextual relevance. Table 3 summarizes construct operationalization, while the complete survey instrument is provided in Appendix B.

#### 3.4.3. Data Analysis

The quantitative data were analysed using PLS-SEM with SmartPLS 4 (Hair et al., 2022). PLS-SEM was considered appropriate because of the exploratory nature of the study, its focus on examining relationships among multiple constructs, and its suitability for prediction-oriented research. The analysis was conducted in two stages. First, the measurement model was evaluated for indicator reliability, internal consistency reliability, and convergent validity. Indicator reliability was evaluated using outer loadings, whereas internal consistency reliability was assessed using Cronbach’s alpha and Composite Reliability (CR). Convergent validity was evaluated using the Average Variance Extracted (AVE). The results of the measurement model assessment are presented in Section 3.5.

Second, the structural model was evaluated to examine the proposed hypothesis. Two separate structural models were estimated using the same sample of 350 founders. The first model examined Strategic Decision Style as the dependent variable, while the second examined Operational Decision Style. In both models, Mind Type, Tactics Type, Domain Experience, Area of Expertise, and Risk Appetite were specified as predictor variables.

The significance of the individual structural relationships was assessed using a bootstrapping procedure with 5000 resamples. Path coefficients, t-values, *p*-values, and confidence intervals were used to assess the significance of each structural path, while the explanatory power of each model was evaluated using the coefficient of determination (R^2^).

To evaluate H1, each structural model was interpreted independently based on the pattern of significant associations, explanatory power (R^2^), and model fit (SRMR). The findings from the two models were then considered collectively to examine whether founder attributes exhibited context-specific patterns of association across strategic and operational decision contexts.

### 3.5. Measurement Model Assessment

The measurement model demonstrated satisfactory reliability and validity. Indicator reliability was confirmed through outer loadings, all of which exceeded the recommended threshold of 0.70 and were statistically significant (*p* < 0.001), indicating strong relationships between the indicators and their respective constructs. Internal consistency reliability was supported by Composite Reliability (CR) values ranging from 0.910 to 0.968 and Cronbach’s alpha values ranging from 0.896 to 0.934, both exceeding the recommended thresholds of 0.70 (Hair et al., 2022). Convergent validity was established using the Average Variance Extracted (AVE), with all values exceeding 0.80, well above the recommended minimum threshold of 0.50. Together, these results demonstrate that the constructs exhibited high internal consistency and explained a substantial proportion of the variance in their indicators. The reliability and convergent validity results are presented in Table 4.

Discriminant validity was evaluated using the Heterotrait–Monotrait (HTMT) ratio. All HTMT values were below the recommended threshold of 0.85, confirming that the constructs were empirically distinct (Table 5). The SRMR values for the strategic (0.065) and operational (0.046) structural models were both below the recommended threshold of 0.08, indicating acceptable model fit (Henseler et al., 2015).

## 4. Findings

### 4.1. Qualitative Findings

**Figure 2:**
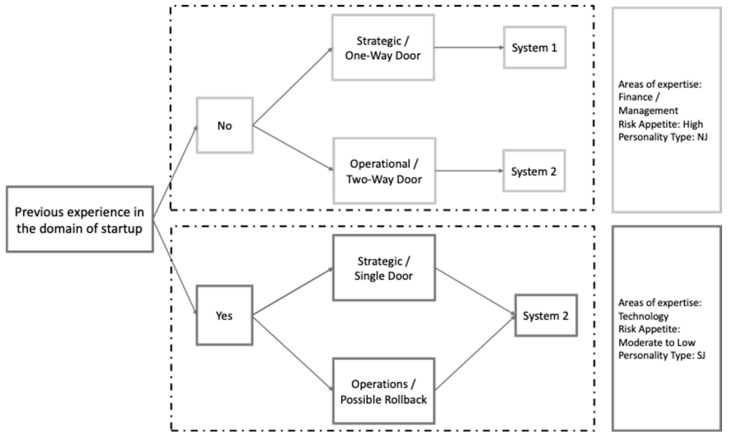
Qualitative Analysis Insights.

Grounded theory analysis of the interview data revealed that founders’ decision-making during the startup execution phase was shaped by the decision context. The interviews consistently distinguished between strategic and operational decisions, suggesting that these represent qualitatively different cognitive contexts (Figure 2). The findings also highlighted the influence of founder attributes, including domain experience and risk appetite, on founders’ reported decision-making approaches.

#### 4.1.1. Decision Types

Across the interviews, founders consistently distinguished between two broad types of decisions during the startup execution phase: strategic and operational decisions. Strategic decisions were frequently described as “one-way door decisions” because they involved high-impact, long-term, and often irreversible choices such as market entry, scaling strategy, and funding decisions. As one founder explained: “Deciding to enter East Zone (new zone) was a strategic decision because it required committing time, people, and capital. Once we expanded, walking back was neither easy nor cost-effective.” (Founder, HealthTech—New Delhi, India).

In contrast, operational decisions were commonly described as “two-way door decisions” because they involved lower-stakes, short-term, and generally reversible choices related to day-to-day coordination and execution. For example, one founder stated: “For me, managerial hiring or adding incremental product features are operational decisions. If something doesn’t work, we can usually adjust and course-correct quickly.” (Founder, SaaS—Bengaluru).

These accounts consistently highlighted that founders perceived strategic and operational decisions as differing in their consequences, reversibility, and time horizon, supporting the distinction between the two decision contexts that informed the study’s conceptual framework.

#### 4.1.2. Strategic Decision-Making Styles

Strategic decisions exhibited considerable variation in self-reported decision-making styles, with domain experience and cognitive orientation emerging as key factors associated with these differences. Founders with limited domain experience generally reported greater reliance on intuitive (System 1) reasoning, emphasizing vision, conviction, and pattern recognition under uncertainty. These founders viewed uncertainty as a source of opportunity and competitive advantage. As one founder explained: “If I had waited for complete information, we would have missed the opportunity. Sometimes you need to make the call based on what feels right and then execute aggressively.” (Founder, EdTech). In contrast, founders with substantial prior domain experience more frequently reported greater reliance on analytical (System 2) reasoning, using scenario analysis, structured risk assessment, and evidence-based evaluation for strategic decisions. Their domain knowledge appeared to provide a foundation for more systematic evaluation before making strategic commitments. As one experienced founder remarked: “Before making a major decision, I usually evaluate different scenarios, assess the downside, and validate assumptions before moving ahead.” (Founder, SaaS).

Overall, the findings indicate that strategic decision-making was characterized by varying degrees of reliance on intuitive and analytical reasoning, with these patterns appearing to be associated with founders’ prior domain experience and cognitive orientation.

#### 4.1.3. Operational Decision-Making Styles

In contrast to strategic decisions, founders reported similar approaches to operational decision-making regardless of their cognitive orientation or prior domain experience. Across the interviews, founders consistently indicated greater reliance on analytical (System 2) reasoning when making operational decisions, emphasizing process adherence, team coordination, and minimizing execution errors. Even founders who expressed a strong preference for intuitive decision-making in strategic contexts reported following structured processes and data monitoring for operational decisions, with intuition serving only a supplementary role. As one founder noted: “I may trust my instincts when making big strategic calls, but operationally we follow processes. If every operational decision is based on intuition, execution becomes inconsistent.” (Founder, EdTech - Mumbai). Overall, these findings indicate that operational decision-making was viewed as being guided primarily by established processes and routines, with founder attributes appearing to play a less prominent role than in strategic decision-making.

#### 4.1.4. Founder Attributes and Contextual Adaptation

A key insight from the qualitative analysis was that the associations between founder attributes and self-reported decision-making styles appeared to depend on the decision context. Founders with intuitive cognitive orientations generally reported greater reliance on System 1 reasoning when making strategic decisions, while adopting System 2 reasoning for operational decisions. This pattern suggests that founders adapt their decision-making approach according to the cognitive demands of the decision they face. Similarly, prior domain experience was associated with strategic decision-making but was less relevant in operational contexts, where both experienced and inexperienced founders emphasized greater reliance on System 2 reasoning. Collectively, these findings indicate that founder attributes exhibited context-dependent patterns of association with decision-making styles, highlighting the importance of considering decision context when examining entrepreneurial decision-making during the startup execution phase.

#### 4.1.5. Summary of Qualitative Insights

The qualitative findings indicate that the association between founder attributes and self-reported decision-making styles depends on the decision context. Two key insights emerged. First, strategic and operational decisions represented distinct cognitive contexts, with founders adapting their reliance on intuitive (System 1) and analytical (System 2) reasoning according to the demands of the decision. Second, founder attributes appeared to be more strongly associated with self-reported decision-making styles in strategic decisions, whereas operational decisions were characterized by more consistent decision-making approaches and greater reliance on analytical reasoning. Collectively, these findings highlight the importance of considering decision context when examining entrepreneurial cognition during the startup execution phase.

### 4.2. Quantitative Results

This section presents results of the PLS-SEM analysis conducted using SmartPLS 4. Consistent with the study design, two separate structural models were estimated using the same sample of founders. The first model examined Strategic Decision Style as the outcome variable, while the second examined Operational Decision Style. The measurement model assessment and structural model analysis are presented below.

#### 4.2.1. Collinearity Assessment

Prior to evaluating the structural model, collinearity among the predictor constructs was assessed using Variance Inflation Factor (VIF) values. All VIF values were below the conservative threshold of 3.3 in both structural models (Table 6), indicating that multicollinearity was not a concern and that the estimated path coefficients could be interpreted reliably.

#### 4.2.2. Structural Model Results

Separate structural models were estimated for the strategic and operational decision contexts to examine the associations between founder attributes and self-reported decision-making styles within each context.

Strategic Decision Context: The structural model for Strategic Decision Style demonstrated moderate explanatory power (R^2^ = 0.497), indicating that the founder attributes collectively explained approximately 49.7% of the variance in self-reported strategic decision-making style. Three predictor variables were significantly associated with Strategic Decision Style (Table 7): Mind Type (β = 0.302, *p* < 0.001) was positively associated with Strategic Decision Style, suggesting that founders with different cognitive orientations reported different strategic decision-making styles. Domain Experience (β = 0.286, *p* < 0.001) was also positively associated with Strategic Decision Style, suggesting that founders reporting less prior industry familiarity were more likely to report intuitive decision-making styles in strategic decision contexts. Risk Appetite (β = 0.240, *p* < 0.001) was similarly significant, suggesting that founders with higher risk tolerance reported different strategic decision-making styles. Area of Expertise (β = −0.067, *p* = 0.098) and Tactics Type (β = −0.042, *p* = 0.332) did not exhibit significant relationships with Strategic Decision Style.

Operational Decision Context: The structural model for operational decision-making style demonstrated markedly lower explanatory power (R^2^ = 0.125), indicating that the founder attributes collectively explained approximately 12.5% of the variance in self-reported operational decision style. Among the predictor variables, only Risk Appetite was significantly associated with Operational Decision Style (β = 0.280, *p* < 0.001. All remaining predictors were non-significant: Mind Type (β = 0.138, *p* = 0.123), Domain Experience (β = −0.050, *p* = 0.591), Area of Expertise (β = 0.042, *p* = 0.436), and Tactics Type (β = −0.059, *p* = 0.356). Results are presented in Table 8.

#### 4.2.3. Comparative Summary

The two structural models (Table 9) revealed different patterns of association between founder attributes and self-reported decision-making styles. In the strategic model, Mind Type, Domain Experience, and Risk Appetite were significantly associated with Strategic Decision Style. In contrast, within the operational model, only Risk Appetite remained significantly associated with Operational Decision Style.

The strategic model explained a larger proportion of the variance in Strategic Decision Style (R^2^ = 0.497) than the operational model explained in Operational Decision Style (R^2^ = 0.125). These findings are consistent with the qualitative results, which indicated that founder attributes played a more prominent role in strategic decision-making, whereas operational decision-making appeared to be guided by established processes, routines, and execution requirements.

Taken together, the qualitative and quantitative findings indicate that founder attributes exhibit context-specific patterns of association with self-reported decision-making styles. As no formal statistical comparison of the two models was performed, these findings should be interpreted as descriptive evidence of context-specific patterns of association.

### 4.3. Integrated Findings

The integration of the qualitative and quantitative findings provides a coherent account of how founder attributes are associated with self-reported decision-making styles during the startup execution phase. Across both phases, the findings indicate context-specific patterns of association across strategic and operational decision contexts.

The qualitative findings indicated that founders adopted different approaches when making strategic and operational decisions. Strategic decision-making was characterized by greater variation in the use of intuitive (System 1) and analytical (System 2) reasoning, particularly among founders with different levels of domain experience and cognitive orientation. In contrast, operational decisions were described as more structured and process-driven, resulting in more consistent decision-making approaches across founders.

The quantitative findings were broadly consistent with these observations. Mind Type, Domain Experience, and Risk Appetite were significantly associated with Strategic Decision Style, whereas only Risk Appetite was significantly associated with Operational Decision Style. In addition, founder attributes accounted for substantially more variance in Strategic Decision Style (R^2^ = 0.497) than in Operational Decision Style (R^2^ = 0.125).

Taken together, the qualitative and quantitative findings indicate that founder attributes exhibit context-specific patterns of association with self-reported decision-making styles. The strategic model demonstrated a broader pattern of significant relationships than the operational model, where only Risk Appetite remained significant. These findings are consistent with H1 and highlight the importance of considering decision context when examining entrepreneurial decision-making during the startup execution phase.

## 5. Discussion

This study examined how founder attributes relate to self-reported decision-making styles during the startup execution phase and explored context-specific patterns of association across strategic and operational decision contexts. Taken together, the qualitative and quantitative findings indicate that entrepreneurial decision-making is context sensitive. Founder attributes exhibited a broader pattern of association with self-reported decision-making styles in the strategic model than in the operational model, highlighting the importance of considering decision context when examining entrepreneurial cognition.

### 5.1. Theoretical Contributions

#### 5.1.1. Applying Dual-Process Theory to Strategic and Operational Decision Contexts

This study contributes to entrepreneurial cognition research by providing a more context-sensitive application of Dual-Process Theory. The findings indicate that founder attributes exhibit context-specific patterns of association with self-reported decision-making styles across strategic and operational decision contexts. Prior applications of Dual-Process Theory in entrepreneurship have often treated intuitive and analytical reasoning as relatively stable individual tendencies or cognitive styles (Hodgkinson & Sadler-Smith, 2018). The present findings suggest that the relevance of System 1 and System 2 reasoning depends on the decision contexts.

Accordingly, the study highlights the importance of considering decision context when applying Dual-Process Theory to entrepreneurial decision-making. In doing so, the study extends the application of Dual-Process Theory by providing a more nuanced understanding of entrepreneurial cognition during the startup execution phase.

#### 5.1.2. Advancing Process-Based Accounts of Entrepreneurial Decision-Making

The findings contribute to entrepreneurship research by supporting a more process-oriented understanding of entrepreneurial decision-making. Prior research has often examined founder attributes as direct predictors of entrepreneurial outcomes (Zhao & Seibert, 2006; Obschonka et al., 2019), implicitly assuming that their influence remains relatively stable across different decision situations. The present findings highlight the importance of decision context by showing that founder attributes exhibit context-specific patterns of association with self-reported decision-making styles. By moving beyond trait-based explanations, the study advances a more process-oriented account of entrepreneurial decision-making during the startup execution phase.

Neither Area of Expertise nor Tactics Type emerged as significant predictors in either the strategic or operational model. One possible explanation is that founders draw on a combination of experiences, situational cues, and contextual information when making decisions, reducing the relative importance of specific functional expertise or preferred decision orientation. These findings suggest that not all founder attributes are equally relevant to founders’ self-reported decision-making styles and highlight the importance of distinguishing between attributes that are theoretically proposed and those that demonstrate empirical relationships with decision-making styles during the startup execution phase.

#### 5.1.3. Reconceptualizing the Startup Execution Phase

The study also contributes by providing a more granular, empirically grounded understanding of the execution phase, a stage that has received comparatively limited theoretical attention. Rather than treating execution as a homogeneous implementation process, the findings suggest that it encompasses qualitatively distinct decision contexts with different cognitive demands on founders. The strategic and operational structural models revealed context-specific patterns of association between founder attributes and self-reported decision-making styles, highlighting the importance of viewing startup execution as a collection of distinct decision situations rather than a single, unified phase. This perspective offers a more nuanced understanding of startup execution and provides a foundation for future research examining how founders navigate different decision contexts as ventures evolve.

### 5.2. Managerial Implications

The findings offer several practical insights for founders, investors, and entrepreneurial support ecosystems. For founders, the results highlight the importance of recognizing that different decision contexts may require different approaches to making decisions. Developing awareness of one’s own decision-making tendencies may help founders reflect on how they approach strategic and operational decisions during the startup execution phase.

For investors and venture capital firms, the findings suggest that trait-based founder assessments (e.g., evaluating experience or risk tolerance in isolation) may provide only a partial understanding of how founders approach important business decisions. Additional insights may be gained by considering how founders adapt their cognitive approach across different decision contexts, particularly in high-stakes strategic situations.

For startup accelerators and training programs, the findings highlight the importance of designing interventions that cultivate context-sensitive decision-making capabilities rather than promoting generic frameworks. Training that encourages founders to reflect on when intuitive (System 1) or analytical (System 2) reasoning may be most appropriate, while recognizing the demands of different decision contexts, may provide greater value than generic decision-making frameworks.

Although the present study does not evaluate decision quality, venture performance, or entrepreneurial outcomes, the findings suggest that understanding how founders adapt their decision-making approach across different contexts may provide useful insights for founder development and entrepreneurial support programs.

### 5.3. Limitations and Future Research

This study has several limitations that should be considered when interpreting the findings and that also provide directions for future research. First, the study was conducted among Indian startup founders, which may limit generalizability of the findings to other institutional, cultural, and regulatory contexts. Future research could examine whether the observed patterns are replicated across diverse entrepreneurial ecosystems.

Second, the study focused on founders of funded or profitable Indian startups. While this enabled the examination of founders with substantial experience during the startup execution phase, it may introduce survivorship bias, as founders whose ventures did not survive or achieve comparable levels of success were not represented. Future research could examine whether similar patterns emerge among unsuccessful ventures or early-stage startups.

Third, decision-making style was captured through self-reported survey data. Consequently, the findings should be interpreted as founders’ self-reported decision-making approaches rather than as direct observations of underlying cognitive processes. Self-reported measures may be influenced by social desirability, perceptual, and retrospective biases. In addition, because both predictor and outcome variables were collected from the same respondents using a single survey instrument, the possibility of common method bias cannot be entirely ruled out. Future research could employ behavioural or experimental designs, such as think-aloud protocols, decision simulations, or experience sampling methods, to capture real-time cognitive processes.

Fourth, while the study focuses on founder attributes and decision type, it does not account for additional contextual factors such as team composition, organizational structure, environmental dynamism, or industry-specific uncertainty. Future research could examine how these factors interact with founder attributes and decision-making styles, particularly in operational contexts, where fewer significant relationships were observed.

Fifth, the quantitative phase employed non-probability quota sampling, which limits the extent to which the findings can be generalized to the broader population of startup founders. While the sampling approach was appropriate given the practical challenges associated with accessing founder populations, future studies could employ probability-based sampling approaches where feasible.

Finally, the present study focused on the association between founder attributes and self-reported decision-making styles and did not examine decision quality, venture performance, or entrepreneurial outcomes. Future research could examine whether different decision-making styles are associated with venture outcomes and formally compare these relationships across strategic and operational decision contexts.

## 6. Conclusions

This study examined how founder attributes are associated with self-reported decision-making styles during the startup execution phase, with particular attention to strategic and operational decision contexts. Using a sequential exploratory mixed-methods design, the study found evidence of context-specific patterns of association between founder attributes and self-reported decision-making styles. Across the qualitative and quantitative phases, founder attributes showed stronger associations with self-reported decision-making styles in strategic contexts than in the operational contexts.

Three key contributions emerge from the study. First, the findings highlight the importance of considering decision context when applying Dual-Process Theory to entrepreneurial decision-making. Second, the study advances a process-based understanding of entrepreneurial decision-making by demonstrating that founder attributes exhibit context-specific patterns of association with self-reported decision-making styles. Third, the study offers a more granular understanding of the startup execution phase by showing that it comprises qualitatively distinct decision contexts rather than a single homogeneous implementation process.

The findings also offer practical insights for founders, investors, and entrepreneurial support systems by highlighting the importance of adapting decision-making approaches to different decision contexts. However, the findings should be interpreted in light of the study’s methodological limitations, including the use of self-reported measures and a non-probability sample of Indian startup founders. As no formal statistical comparison of the two structural models was performed, the findings should be interpreted as descriptive evidence of context-specific patterns of association. Future research could build on this work by examining entrepreneurial decision-making across different institutional and cultural settings, employing behavioural measures of decision-making, and using appropriate statistical techniques to formally compare the relationships between decision-making styles and venture outcomes across strategic and operational decision contexts.

## Figures and Tables

**Table 1:** Sample Demographics Across Qualitative and Quantitative Phases.

Variable	Category	Qualitative (n = 20)		Quantitative (n = 350)	
		Freq.	%	Freq.	%
Prior Domain Exp.	Yes	11	55%	167	48%
	No	9	45%	183	52%
Founder Age	<35 years	8	40%	199	57%
	>35 years	12	60%	151	43%
Gender	Male	17	85%	292	83%
	Female	3	15%	58	17%
Education	Bachelors	8	40%	186	53%
	Masters & Above	12	60%	164	47%
Area of Expertise	Finance	4	20%	63	18%
	Management	6	30%	135	39%
	Others	10	50%	152	43%
Venture Age	2–5 years	9	45%	193	55%
	5+ years	11	55%	157	45%
Venture Type	B2B	10	50%	133	38%
	B2C	8	40%	165	47%
	B2B2C	2	10%	52	15%
Venture Category	Product	8	40%	157	45%
	Service	12	60%	193	55%
Venture Size	Small (<50)	8	40%	137	39%
	Medium (50–200)	6	30%	119	34%
	Large (>200)	6	30%	94	27%

**Table 2:** Sample Coding Progression Matrix.

Representative Quote	Open Code	Axial Category	Selective Theme
“I transitioned from finance to healthtech.”	No Prior Domain Experience	Domain Experience	Founder Attributes
“I’m willing to take big risks for big rewards.”	High Risk Appetite	Entrepreneurial Attributes	Founder Attributes
“These decisions are difficult to reverse and influence the future of the business.”	Strategic Decision	Decision Type	Decision Context
“I go by intuition, make fast decisions, and move on.”	Intuitive Processing	Decision Style	Decision-Making Style
“I use data and draw on past experience before deciding.”	Analytical Processing	Decision Style	Decision-Making Style

**Table 3:** Construct Operationalization.

Construct	Items	Example Item	Source
Domain Experience	1	“I had no prior experience in my startup’s industry/domain before launching it.”	Developed for this study
Area of Expertise	1	“Before starting my venture, I possessed expertise in finance or management.”	Developed for this study
Risk Appetite	3	“I am comfortable committing significant resources to an opportunity even when outcomes are uncertain.”	Adapted from Zhao et al. (2010)
Mind Type	2	“I often rely on my gut feeling or intuition when making important decisions.”	Adapted from Allinson and Hayes (1996)
Tactics Type	2	“I prefer having a clear and structured plan before execution rather than improvising.”	Adapted from Scott and Bruce (1995)
Strategic Decision Style	2	“When making strategic decisions, I rely primarily on intuition and experience.”	Developed for this study
Operational Decision Style	2	“When making operational decisions, I rely primarily on intuition and experience.”	Developed for this study

**Table 4:** Construct Reliability and Validity.

Construct	Indicator	Outer Loading	Composite Reliability (CR)	AVE	Cronbach’s Alpha
Mind Type	MT1	0.968	0.967	0.937	0.932
	MT2	0.968			
Risk Appetite	RA1	0.907	0.935	0.827	0.896
	RA2	0.913			
	RA3	0.908			
Tactics Type	TT1	0.823	0.910	0.910	0.926
	TT2	0.997			
Strategic Decision Style	SDS3	0.967	0.968	0.938	0.934
	SDS4	0.970			
Operational Decision Style	ODS3	0.952	0.956	0.929	0.932
	ODS4	0.962			

Note. All outer loadings significant at *p* < 0.001 (bootstrapping, 5000 subsamples). CR = Composite Reliability; AVE = Average Variance Extracted.

**Table 5:** Discriminant Validity Assessment (HTMT).

Construct	AOE	DE	MT	RA	TT	SDS	ODS
Area of Expertise (AOE)	-						
Domain Experience (DE)	0.190	-					
Mind Type (MT)	0.258	0.794	-				
Risk Appetite (RA)	0.174	0.587	0.521	-			
Tactics Type (TT)	0.120	0.019	0.120	0.143	-		
Strategic Decision Style (SDS)	0.110	0.662	0.661	0.588	0.026	-	
Operational Decision Style (ODS)	0.126	0.218	0.246	0.355	0.115	-	

Note. Strategic Decision Style (SDS) and Operational Decision Style (ODS) were estimated in separate structural models; therefore, HTMT values between SDS and ODS were not computed.

**Table 6:** Variance Inflation Factor (VIF) Statistics.

Predictor Variable	VIF
Strategic Decision Context	
Area of Expertise → Strategic Decision Style	1.076
Domain Experience → Strategic Decision Style	2.745
Mind Type → Strategic Decision Style	2.542
Risk Appetite → Strategic Decision Style	1.499
Tactics Type → Strategic Decision Style	1.038
Operational Decision Context	
Area of Expertise → Operational Decision Style	1.092
Domain Experience → Operational Decision Style	2.736
Mind Type → Operational Decision Style	2.613
Risk Appetite → Operational Decision Style	1.507
Tactics Type → Operational Decision Style	1.087

Note. All VIF values are below the recommended threshold of 3.3, indicating no significant multicollinearity concerns.

**Table 7:** Structural Model Results—Strategic Decision Style.

Path	Β	t-Value	*p*-Value	Result
Area of Expertise → Strategic Decision Style	−0.067	1.656	0.098	Not Significant
Domain Experience → Strategic Decision Style	0.286	3.881	<0.001	Significant ***
Mind Type → Strategic Decision Style	0.302	4.167	<0.001	Significant ***
Risk Appetite → Strategic Decision Style	0.240	5.778	<0.001	Significant ***
Tactics Type → Strategic Decision Style	−0.042	0.971	0.332	Not Significant

Note. *** *p* < 0.001 (bootstrapping, 5000 subsamples). β = standardized path coefficient.

**Table 8:** Structural Model Results—Operational Decision Style.

Path	Β	t-Value	*p*-Value	Result
Area of Expertise → Operational Decision Style	0.042	0.778	0.436	Not Significant
Domain Experience → Operational Decision Style	−0.050	0.538	0.591	Not Significant
Mind Type → Operational Decision Style	0.138	1.541	0.123	Not Significant
Risk Appetite → Operational Decision Style	0.280	5.683	<0.001	Significant ***
Tactics Type → Operational Decision Style	−0.059	0.922	0.356	Not Significant

Note. *** *p* < 0.001 (bootstrapping, 5000 subsamples). β = standardized path coefficient.

**Table 9:** Comparative Structural Model Results: Strategic versus Operational Decision Contexts.

Predictor	Strategic β (*p*-Value)	Operational β (*p*-Value)	Interpretation
Mind Type	0.302 (<0.001)	0.138 (=0.123)	Significant only in the strategic decision context
Risk Appetite	0.240 (<0.001)	0.280 (<0.001)	Significant across both decision contexts
Domain Experience	0.286 (<0.001)	−0.050 (=0.591)	Significant only in the strategic decision context
Area of Expertise	−0.067 (=0.098)	0.042 (=0.436)	Not significant in either context
Tactics Type	−0.042 (=0.332)	−0.059 (=0.356)	Not significant in either context

## Data Availability

The data, survey instrument, analysis files, and supporting materials used in this study are available in an Open Science Framework (OSF) repository. During the peer-review process, the repository is accessible through an anonymous view-only link provided to the editor and reviewers. Upon publication, the repository will be made publicly accessible through its permanent OSF link.

## Appendix A. Semi-Structured Interview Guide

Section A: Founder Profile

1. Name (optional/coded ID)
2. Startup Name (optional/coded ID)
3. Year of founding
4. Age at the time of starting the venture
5. Gender
6. Educational background
7. Area of expertise/specialization
8. Nature of venture (Product/Service/Hybrid)
9. Business model (B2B/B2C/B2B2C)
10. Prior industry/domain experience
11. Years of experience in the venture domain
12. Prior entrepreneurial experience
13. Current team size
14. Funding status (Bootstrapped/Funded)

Section B: Entrepreneurial Motivation and Background

15. Can you describe the key trigger or motivation that led you to start your venture?
16. How did your educational and professional background prepare you for starting this venture?
17. Can you describe situations where your expertise helped you make important business decisions?
18. Have there been situations where a lack of experience or expertise created challenges in decision-making?

Section C: Risk Appetite

19. How would you describe your approach to risk as an entrepreneur?
20. Can you share an example of a decision that involved significant uncertainty or risk?
21. What factors influence your willingness to take risks?

*Probe: Comfort with ambiguity, Resource commitment, Calculated vs intuitive risk-taking, Risk mitigation approaches*

Section D: Decision-Making Process

22. Can you walk me through how you typically make important business decisions as a founder?
23. What factors influence your decisions? (*Probe role of intuition, analysis, experience, and decision context in decision-making*)
24. How do you make decisions when information is incomplete or uncertain?
25. Can you describe two recent important decisions you have made and explain how you arrived at them?

*Probe: Level of uncertainty, Use of data, Role of intuition, Alternatives considered, Speed of decision*

Section E: Decision-Making Style & Reflection

26. Do you find that your approach changes depending on the type of decision being made? Please provide examples.
27. Are there situations where you rely more on intuition than analysis? What drives that choice?
28. Are there situations where you rely more on analysis than intuition? What drives that choice?
29. How has your decision-making approach evolved over time?

*Probe: Nature of the decision, Time horizon*

Section F: Cognitive Preferences

30. When evaluating a new situation or problem, do you tend to focus more on immediate facts and practical realities, or on future possibilities and patterns? Can you provide an example?
31. Do you rely more on concrete information that is directly available, or do you often draw conclusions based on patterns, connections, and possibilities? Why?

*Probe: Attention to facts versus possibilities, Practicality versus exploration, Experience versus future orientation, Detail orientation versus big-picture thinking*

32. When managing important decisions, do you prefer having a clear plan and defined milestones, or do you prefer keeping options open as circumstances evolve? Why?
33. How do you respond when unexpected opportunities or challenges require you to modify existing plans?

*Probe: Preference for structure, Flexibility and adaptability, Closure versus exploration, Planning versus improvisation*

## Appendix B. Survey Questionnaire

Section A: Founder Profile

1. Your Name (optional/coded ID):
2. Startup Name (optional/coded ID):
3. Year Started:
4. Age at the time of starting the venture:
5. Gender:
6. Educational background:
7. Domain/Area of the startup:
8. Nature of venture (Product/Service/Hybrid):
9. Business model (B2B/B2C/B2B2C):
10. Current team size (<50, 50–200, >200):
11. Funding status (Bootstrapped/Funded):
12. Years of experience prior to starting the venture:
13. Years of experience in the domain of the startup:
14. Area of expertise (formal education, professional experience, or specialized training):
  □ Finance/Management □ Technology/Engineering □ Sales/Marketing □ Operations
  □ Healthcare/Pharma. □ Other __________

*Please indicate how much you agree with each of the following statements as they apply to you as a founder (Scale: Strongly Disagree, Disagree, Neither agree nor disagree, Agree, Strongly agree).*

15. I had no prior experience in my startup’s industry/domain before launching it.
16. Before starting my venture, I possessed expertise in finance or management (formal education, professional experience, or specialized training).

Section B: Risk Appetite

17. I am comfortable committing significant resources to an opportunity even when outcomes are uncertain.
18. I am willing to make important business decisions despite incomplete information.
19. I actively pursue opportunities that involve substantial uncertainty and risk.

Section C: Mind Type

20. I often rely on my gut feeling or intuition when making important decisions.
21. I frequently recognize patterns or opportunities before they become obvious to others.
22. I tend to focus on future possibilities rather than immediate realities when evaluating opportunities.
23. I often trust my instincts when assessing new business opportunities.

Section D: Tactics Type

24. I prefer having a clear and structured plan before execution rather than improvising.
25. Once a course of action is decided, I prefer focusing on execution rather than continuing to explore alternatives.
26. I prefer clearly defined goals and milestones when pursuing business objectives.
27. I prefer making decisions and moving forward rather than keeping multiple options open.

Section E: Strategic Decision-Making Style
*Strategic decisions refer to major decisions such as entering new markets, launching new products, raising capital, partnerships, acquisitions, or changing business models.*

28. When making strategic decisions, I rely primarily on structured analysis and data.
29. I carefully evaluate multiple alternatives before making strategic decisions.
30. When making strategic decisions, I rely primarily on intuition and experience.
31. I prefer to act instinctively rather than overanalyse strategic decisions.

Section F: Operational Decision-Making Style
*Operational decisions refer to day-to-day decisions related to execution, people, processes, customers, quality, and business operations.*

32. When making operational decisions, I rely primarily on structured analysis and data.
33. I carefully evaluate multiple alternatives before making operational decisions.
34. When making operational decisions, I rely primarily on intuition and experience.
35. I prefer to act instinctively rather than overanalyse operational decisions.
